# Acknowledgement to Reviewers of *Pharmaceuticals* in 2014

**DOI:** 10.3390/ph8010038

**Published:** 2015-01-08

**Authors:** Pharmaceuticals Editorial Office

**Affiliations:** MDPI AG, Klybeckstrasse 64, CH-4057 Basel, Switzerland

The editors of *Pharmaceuticals* would like to express their sincere gratitude to the following reviewers for assessing manuscripts in 2014:

Albericio, Fernando
Apfel, Christian C.
Behe, Martin
Bergheim, Ina
Broder, M.
Carroll, F. Ivy
Catts, Vibeke
Chapman, Eli
Chen, Kai
Chen, S.
Chen, Xuanmao
Chiang, Chiao-Hsi
Chong, Parkson Lee-gau
Dahan, Albert
Dehaye, Jean-Paul
Denkova, Antonia
Deslouches, Berthony
Deuther-Conrad, Winnie
Dietrich, André
Dilda, Pierre
D'Mello, Santosh
Dumont, Rebecca A.
Edafioghoa, Ivan O.
Ellerby, Lisa
Entelis, Nina
Fagerlund, Malin Jonsson
Fenn, Ashley M.
Francia, Giulio
Gafencu, Anca
Gallagher, John F.
Grudzinski, Joseph J.
Gueimonde, Miguel
Guetschow, Michael
Guglielmetti, Simone
Gynther, Mikko
Hassan, Hosni
Himmelsbach, Markus
Hockley, Brian
Hohaus, Stefan
Hudecek, Michael
Hwang, William Ying Khee
Inohara, N.
Irato, Paola
Johnston, Graham A.R.
Kazak, Lawrence
Kiesewetter, Dale O.
Kogut, Michael H.
Komatsuzawa, Hitoshi
Koulen, Peter
La Thangue, Nicholas B.
Leboeuf, Celine
Lehrer, Robert I.
Li, Congjun
Liekens, Sandra
Lin, Jun
Lipkowski, Jacek
Lorenzo, Alibardi
Luo, Jian-Hua
Mäcke, Helmut
Marx, Florentine
Mattoo, Autar
Maus, Marcela V.
McClain, Craig
Mcfarland, Lynne V.
Metz-Boutigue, Marie-Hélène
Meyer, Geerd-J.
Michailidis, G.
Min, Kyung Rok
Mishra, Vinod Kumar
Mohamedali, Khalid
Morales, Juan C.
Mudge, David W.
Muncuoglu, Kosta Muncuoglu
Nanovskaya, Tatiana N.
Nayduch, Dana
Nencka, Radim
Norata, Danilo
O'Flaherty, Sarah
Oratz, Ruth
Palmer Jr, Robert J.
Pandey, Kailash N.
Patten, Glen S.
Pepeu, Giancarlo
Pillay, Viness
Plummer, Sue
Pouget, Jean-Pierre
Qin, Wenmin
Rocque, Gabrielle B.
Rogozhin, E.A.
Sainlos, Matthieu
Salvatore, Francesco
Schibli, Roger
Schröder, Jens-michael
Si, Hongwei
Simerska, Pavla
Smith, Ian
Stessman, Holly A.F.
Suzuki, Nao
Taïeb, David
Tao, Feng
Tarantino, Giovanni
Verbruggen, Alfons
Villalobos, Carlos
Voss, Logan
Wade, John
Wang, Jun
Wei, Yufeng
Wells, Geoffrey
Whitehouse, Michael
Xie, Bin
Xu, Xiaochun
Yan, Guang
Yan, Ru
Yokoyama, Shinji
Yuan, Ze-Chun
Zasloff, Michael
Zech, S.G.
Zhang, Junbo
Zhang, Ming-Rong

